# Consumption of Clarified Grapefruit Juice Ameliorates High-Fat Diet Induced Insulin Resistance and Weight Gain in Mice

**DOI:** 10.1371/journal.pone.0108408

**Published:** 2014-10-08

**Authors:** Rostislav Chudnovskiy, Airlia Thompson, Kevin Tharp, Marc Hellerstein, Joseph L. Napoli, Andreas Stahl

**Affiliations:** Department of Nutritional Sciences and Toxicology, Graduate Program in Metabolic Biology, University of California, Berkeley, California, United States of America; Nihon University School of Medicine, Japan

## Abstract

To determine the metabolic effects of grapefruit juice consumption we established a model in which C57Bl/6 mice drank 25–50% sweetened GFJ, clarified of larger insoluble particles by centrifugation (cGFJ), *ad libitum* as their sole source of liquid or isocaloric and sweetened water. cGFJ and control groups consumed similar amounts of liquids and calories. Mice fed a high-fat diet and cGFJ experienced a 18.4% decrease in weight, a 13–17% decrease in fasting blood glucose, a three-fold decrease in fasting serum insulin, and a 38% decrease in liver triacylglycerol values, compared to controls. Mice fed a low-fat diet that drank cGFJ experienced a two-fold decrease in fasting insulin, but not the other outcomes observed with the high-fat diet. cGFJ consumption decreased blood glucose to a similar extent as the commonly used anti-diabetic drug metformin. Introduction of cGFJ after onset of diet-induced obesity also reduced weight and blood glucose. A bioactive compound in cGFJ, naringin, reduced blood glucose and improved insulin tolerance, but did not ameliorate weight gain. These data from a well-controlled animal study indicate that GFJ contains more than one health-promoting neutraceutical, and warrant further studies of GFJ effects in the context of obesity and/or the western diet.

## Introduction

The unabated increase in incidence of obesity and obesity-associated disorders, particularly type-2 diabetes, continues to present monumental challenges to health [1]. Dietary modification, including use of neutraceuticals, offer promising approaches to ameliorate obesity and its effects, and to increase health-span. Grapefruit juice (GFJ) is relatively rich in nutrients, including vitamins and minerals, and has fewer calories than other many juices [2], [3]. Putative health and weight-loss promoting effects of grapefruit or GFJ consumption have been popularized, but mostly in context of a hypocaloric diet, e.g. the “Hollywood diet”, which limits caloric intake to as low as 3349 kJ per day. Relatively few human studies have examined the effects of grapefruit or GFJ consumption per se on metabolism in well-controlled experiments, and these have produced intriguing, but contradictory results. Fujioka et al. reported that consumption of GFJ, whole grapefruit, or “grapefruit pills” led to weight loss and improved insulin sensitivity [4]. In contrast, Silver et al. reported that grapefruit or GFJ consumption had no significant effects on metabolic variables, except for a modest increase in HDL, in obese participants fed a restricted calorie diet [5].

Studies in animals have used GFJ administered *ad libitum* or have focused on one bioactive component, such as the flavonoid naringin, which contributes to GFJ's bitter taste, or on its aglycone, naringenin. These studies did not address differences in water consumption between treatment and control groups, and produced varied results. Mice are adverse to the bitter taste of GFJ and naringin, which could cause dehydration, reluctance to eat and weight loss independent of metabolic effects. For example, Jung et al. reported that naringin added to food decreases blood glucose in *db/db* mice, but has no effect on body weight [6]. Kannappan and Anuradha reported that naringin affects nutrient and energy metabolism, as well as insulin sensitivity [7]. Pu et al. reported that naringin added to the drinking water of mice fed a high-fat diet (HFD) leads to weight loss, decreased blood glucose, and improved insulin sensitivity [8]. Studies focusing solely on naringin overlook the complex phytochemical composition of GFJ with many potential nutraceutical compounds including bergamottin—a cytochrome P450 inhibitor with potential anti-tumor effects [9].

Other research has focused on GFJ and/or naringin-drug interactions [10], [11]. Naringin has been identified as an inhibitor of Cyp3A4 and organic anion transport protein, which mediate drug catabolism and enterocyte export, respectively. Combined effects of these two have been revealed as a mechanism whereby GFJ can alter intestinal first pass clearance of various drugs, such as statins [10], [12].

We report a model in which mice consumed centrifugation clarified GFJ (cGFJ) *ad libitum* at rates comparable to liquid consumption of control groups. cGFJ consumption did not modify food intake or absorption. In mice fed a HFD, cGFJ decreased the rate of weight gain, hepatic triacylglycerol accumulation, and fasting blood glucose, and improved insulin sensitivity. In mice fed a LFD, cGFJ consumption produced a two-fold decrease in fasting insulin. These data rely on a well-controlled animal model to reveal that GFJ consumption has health-promoting effects, and these effects are mediated by compounds in addition to naringin.

## Materials and Methods

### GFJ preparation

GFJ was squeezed from fresh California Ruby Red grapefruit provided by the California Grapefruit Growers Cooperative, centrifuged at 10,400×g for 10 min at 4°C to remove pulp, amended with 0.15% saccharin (w/v), divided into 25 ml aliquots, and stored at -20°C [13], [14]. The pH of this clarified preparation (cGFJ) was 3.5, compared to 5.5 for the sweetened water used as control. We determined that the caloric content of the cGFJ was 1335 J/ml by bomb calorimetry of a lyophilized sample as previously described [15]. Control mice were given water with 4% glucose (w/v) and 0.15% saccharin (hereafter called control or control water), so that all groups consumed isocaloric liquids with the same amount of saccharin.

### Animals and diets

Procedures were approved by the University of California-Berkeley Animal Care and Use Committee and were done according to AAALAC guidelines. Four-week-old male C57BL/6J mice were purchased from Jackson Laboratories (catalog # 000664). Mice were housed individually and were fed purified diets upon arrival (unless noted otherwise) with either 10% fat (LFD) (Research Diets Cat. # D12450B) or 60% fat (HFD) (Research Diets Cat. #D12492). Any stress induced by housing mice in isolation was normalized by equivalent and concurrent treatment of mice in each experiment.

Mice were weighed three times per week. Food consumption was monitored twice per week. Mice were divided randomly into groups of six (unless noted otherwise): controls (water with 4% glucose and 0.15% saccharin); 50% cGFJ (50% cGFJ/water with 0.15% saccharin); 25% cGFJ (25% cGFJ/water with 4% glucose and 0.15% saccharin); naringin (0.72 mg/day in water with 4% glucose and 0.15% saccharin); metformin (7.5 mg/day metformin with 4% glucose and 0.15% saccharin); metformin + cGFJ (7.5 mg/day metformin with 0.15% saccharin in 50% cGFJ). Liquids were given in volumetric bottles (Med Associates, cat # PHM-127-15) to quantify consumption and were replaced daily.

### Blood glucose

Glucose was measured Monday, Wednesday, and Friday between 9 and 11 AM with a NovaMax blood glucose monitor in blood from a tail prick (AmericanDiabetesWholesale). Glucometer values were corrected using a glucose enzymatic assay kit (Sigma, cat # GAHK20-1KT).

### Glucose (GTT), insulin (ITT), and pyruvate tolerance tests (PTT)

For the GTT, mice were fasted overnight and injected i.p. with 0.2 ml of glucose in sterile water to deliver 2 g/kg glucose. For the ITT, mice were fasted 4 hr and injected i.p. with 0.75 units of insulin/kg. For the PTT, mice were fasted overnight and injected i.p. with 0.2 ml of pyruvate in sterile PBS to deliver 2 g/kg.

### Insulin ELISA

Insulin concentrations were determined with a high-range insulin ELISA kit (ALPCO cat# 80-INSMSH-E01, E10) in blood taken retro-orbitally after an overnight fast. Mice were allowed access to food 4 hr and were re-sampled.

### Protein and triacylglycerol (TG) concentrations of organ lysates

Protein concentrations were assayed with a BCA protein assay kit (Thermo Scientific cat# 23227). TG concentrations were assayed with the Infinity TG kit (Thermo Scientific cat# TR2241).

### Immunohistochemistry

Livers were fixed 1 hr at 4°C with 4% paraformaldahyde, and were incubated overnight at 4°C with a cryopreservation medium of 30% sucrose, 20% Optimal Cutting Temperature medium (VWR cat# 25608-930), and 50% Superblock consisting of Block plus 2% normal donkey serum. Block consisted of 50 ml 10× Hanks balanced salt solution, 50 ml fetal calf serum, 5 g bovine serum albumin, and 0.25 g saponin in 500 ml. Blocks were sectioned into 8 µm strips at −23°C. Sections were stained 1 hr at room temperature with a nonpolar BODIPY probe (Molecular Probes cat# D-3922). Slides were mounted with DAPI/glycerol mounting medium (Life Technologies cat# S36938) and stored at −20°C until imaging.

### Real-time PCR

Real-Time PCR was performed using the TaqMan Universal Master Mix II (Applied Biosystems). Primers were purchased from Integrated DNA Technologies (Table 1).

**Table 1:** Sequences of primers and probes used for real-time PCR.

Gene	Primer 1	Primer 2	Probe
OATP	GATGCTTCAAAGTCCAGTGAC	CACTCCCTCACTTCATCTCAG	56_FAM/CTATGACCA/ZEN/CAGCAGCTCCGACAA/3IABkFQ
SHP	CAAGGAGTATGCGTACCTGAAG	TCCAAGACTTCACACAGTGC	56_FAM/ATCCTCTTC/ZEN/AACCCAGATGTGCCAG/3IABkFQ
Cyp7A1	CACCATTCCTGCAACCTTCT	TCTGTAATGCTCCATTCACTTCT	56_FAM/TGCTTTCAT/ZEN/TGCTTCAGGGCTCCT/3IABkFQ
GCG	GACTCCCTCTGTCTACACCT	CACCAGCATTATAAGCAATCCAG	56_FAM/TTTCTGCCT/ZEN/TGTGAGCCTGAGCT/3IABkFQ
GAPDH	AATGGTGAAGGTCGGTGTG	GTGGAGTCATACTGGAACATGTAG	56_FAM/TGCAAATGG/ZEN/CAGCCCTGGTG/3IABkFQ
FAS	AGTTTGTATTGCTGGTTGCTG	GACTTCTACTGCGATTCTCCTG	56_FAM/TGCGCCTCG/ZEN/TGTGAACATGGA/3IABkFQ
SREBP1C	CGAGATGTGCGAACTGGAC	GTCACTGTCTTGGTTGTTGATG	56_FAM/TGGAGCATG/ZEN/TCTTCGATGTCGTTCAA/3IABkFQ
FGF15	TCTGAAGACGATTGCCATCAAG	AGCCTAAACAGTCCATTTCCTC	56_FAM/ATCAGCCCG/ZEN/TATATCTTGCCGTCC/3IABkFQ
FGF21	GGGATGGGTCAGGTTCAGA	CAGCCTTAGTGTCTTCTCAGC	56_FAM/TCAACACAG/ZEN/GAGAAACAGCCATTCACT/3IABkFQ
PGC1a	CTGCATTCATTGTAGCTGAGC	AGTCCTTCCTCCATGCCT	56_FAM/TGCCAGTAA/ZEN/GAGCTTCTTAAGTAGAGACGG/3IABkFQ
PEPCK	GGATGTCGGAAGAGGACTTTG	GCGAGTCTGTCAGTTCAATACC	56_FAM/CATACATGG/ZEN/TGCGGCCTTTCATGC/3IABkFQ
G6P	GACACCGACTACTACAGCAAC	GACCATAACATAGTATACACCTGCT	56_FAM/CTGTGAGAC/ZEN/CGGACCAGGAAGTC/3IABkFQ

### Western blotting

Livers were homogenized with a Polytron PT2100 in radio immunoprecipitation lysis buffer containing protease and phosphatase inhibitors (Sigma cat# P8340 and cat# P5726) and centrifuged 5 min at 3220×g. Protein (50 µg) was loaded onto a 4–20% Tris-glycine gel. Antibodies were purchased from Cell Signaling. Signals were quantified with a LI-COR Odyssey gel analysis system and normalized to β-tubulin.

### Absorption assays

At 4-weeks-old, mice (7 per group) were fed a HFD for 2 wk while drinking 50% cGFJ or control water *ad libitum*. Mice were fasted overnight and gavaged with 740 kBq [^14^C]oleate in 200 µL olive oil, or 740 kBq [^3^H]2-deoxy-D-glucose in 200 µl sterile PBS containing 2.5 g/kg glucose, or 740 kBq [^14^C]taurocholic acid in 500 µM taurocholic acid in sterile water. Blood was taken retro-orbitaly 15, 60, 120, 180, and 240 min after dosing. Radioactivity was measured in 10 µL serum.

### Indirect calorimetry

Mice were assayed individually by indirect calorimetry (Columbus Instruments, Columbus Ohio, US) during a fast or after fasting 7 hr and re-feeding 1.1 g of the HFD, followed by fasting overnight. Experimental analyses were started between 3–4 PM and continued for ∼23 hr. Activity was monitored in 10 min intervals.

### Fatty acid concentrations and synthesis

Total liver FA concentrations (C16:0, C16:1, C18:0, C18:1 and C18:2) were determined by gas chromatography-flame ionization detection [15]. Palmitate synthesis was measured by analysis of stable isotope incorporation. On day 0 mice were injected i.p. with 100% D_2_O (Sigma cat # 151890) containing 0.9% NaCl (0.35 ml/g body weight). Mice were given 8% D_2_O in their drinking solutions for 17 d. Deuterium incorporation into serum and liver was determined by GC/MS analysis [16]–[18]. Palmitate synthesis was calculated as the fraction of newly synthesized palmitate × total mg palmitate.

### Statistics

Statistical analysis was performed as described in the figure legends. Data are means ± SE. Statistical significance was determined by two-tailed, unpaired t-tests.

## Results

### Isocaloric cGFJ administration

**Figure 1:**
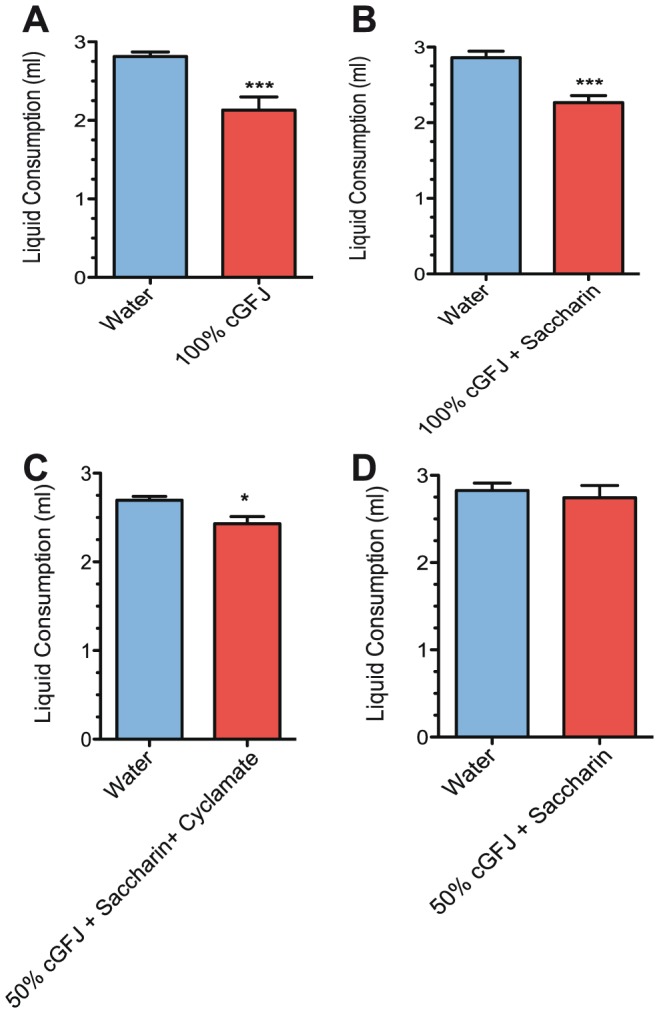
Effects of cGFJ/sweetener on liquid consumption. Mice were given 100% cGFJ, 100% cGFJ +0.15% saccharin, 50% cGFJ +0.15% saccharin +1.5% cyclamate or 50% cGFJ +0.15% saccharin as their sole liquids: A) liquid consumption, ***P = 0.0007; B) liquid consumption, ***P<0.0001; C) liquid consumption, *P<0.02; D) liquid consumption, *P*>0.6. Statistical significance was determined by two-tailed, unpaired t-tests.

Based on average daily liquid consumption, mice were adverse to drinking unsweetened 100% cGFJ, sweetened 100% cGFJ, or saccharin/cyclamate sweetened 50% cGFJ/water (v/v) (Fig. 1A-C). In contrast, cGFJ consumption was comparable to control-group liquid consumption when mice were given 50% GFJ sweetened with 0.15% saccharin (Fig. 1D).

### Impact of GFJ on food consumption, absorption, and energy expenditure

**Figure 2:**
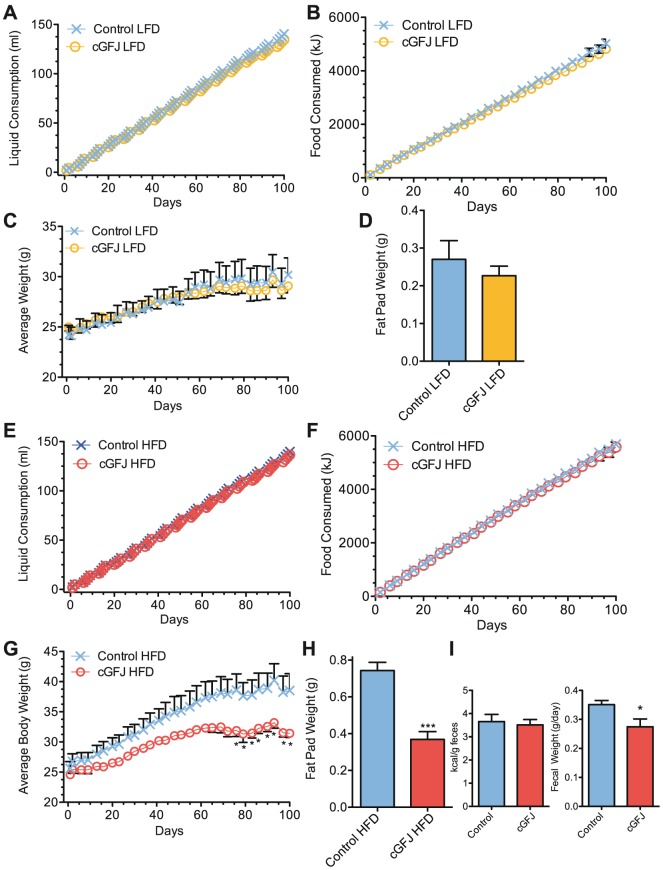
Effects of cGFJ on liquid and food intake, and weight. Mice were fed a LFD or HFD and 50% cGFJ for 100 days, starting from weaning (day 0) at 4 wk old. LFD: A) cumulative liquid consumption; B) cumulative food consumption; C) total body weights; D) intra-abdominal fat pad weight. HFD: E) cumulative liquid consumption; F) cumulative food consumption, *P<0.05; G) total body weights, ***P = 0.0001; H) intra-abdominal fat pad weight, *P<0.05*; I) caloric content of feces of cGFJ and control water treated mice fed a HFD for 100 days, *P*>0.7 and fecal weight of mice collected over 24 hr at the end of 106 days of treatment, *P*>0.03 for GFJ. A two-tailed, unpaired t-test was used to determine statistical significance.

Mice were fed a either a LFD or a HFD for 100 d with access to “control water” (see Material and Methods) or 50% cGFJ as their sole sources of liquids. cGFJ intake did not affect average daily nor cumulative food consumption during a LFD (Fig. 2A, B). Total liquid consumed by LFD-fed mice was 141±1.1 ml water vs. 135±0.5 ml 50% cGFJ (*P*<0.05). This 6 ml difference in liquid consumed over 100 days represents an energy intake difference of <8 kJ or ∼0.002% of total caloric intake. Total calories consumed by the cGFJ group were 4822±83 kJ vs. 5023±163 kJ for controls (*P*>0.05). No differences occurred in weight between the GFJ and control (Fig. 2C). Consistent with similar weights, no differences occurred in epididymal fat pads for LFD-fed mice (Fig. 2D).

cGFJ intake also did not affect average daily nor cumulative liquid and food consumption during feeding a HFD (Fig. 2E, F). The 50% GFJ group consumed 137±0.5 ml vs. 140±2.2 ml by controls (*P*>0.05). Cumulative food consumption was 5580±193 kJ for the GFJ group vs. 5684±155 kJ for controls (*P*>0.05). In contrast to the LFD-fed mice, the HFD-fed mice with access to 50% GFJ weighed 18.4% less than controls at the end of the 100 d: 31.4±0.7 g *vs.* 38.5±2.8 g, *P*<0.05 (Fig. 2G). Body weight trended lower within 15 d after initiating GFJ access and became statistically significant by day 78. Epididymal fat pads of the cGFJ group weighed 50% less than those of the control group (Fig. 2H).

The caloric value of feces collected over the final 24 hr of the 100-day-study from the cGFJ group was similar to controls, as measured by bomb calorimetry, even though average daily fecal mass was ∼23% lower in the cGFJ group (Fig. 2I).

After two weeks feeding a HFD, radiolabeled metabolites in serum (AUC) of mice gavaged with [^3^H]glucose, [^14^C]oleic acid or [^14^C]taurocholic acid did not differ between cGFJ and controls during a 240 min assay (data not shown).

Indirect calorimetry of fasted mice revealed no significant differences in 24 hr energy expenditure (VO_2_ and VCO_2_), substrate use (respiratory exchange ratio), heat production or activity between the HFD-fed GFJ and control groups (data not shown).

### cGFJ improves metabolic variables

**Figure 3:**
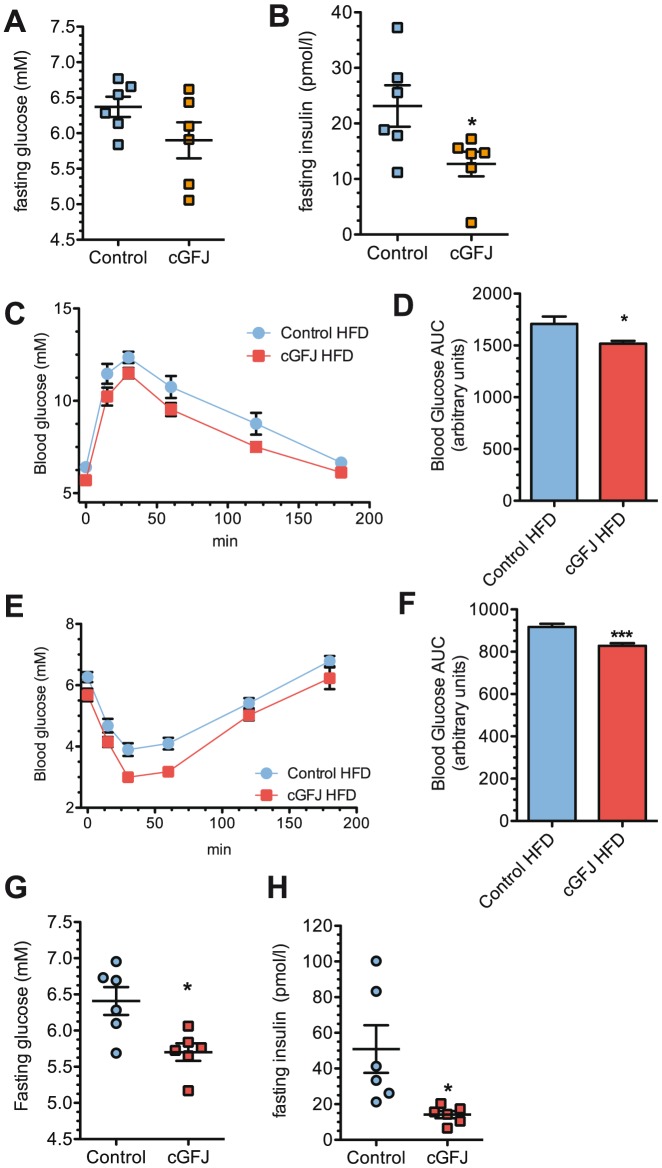
cGFJ effects on blood glucose and insulin sensitivity. Mice were treated as described in the legend of Fig. 2. A, B) values at the end of 100 d LFD: A, fasting blood glucose; B) fasting serum insulin, *P<0.04; C, D) GTT and AUC of mice fed a HFD at week 13, *P<0.04; E, F) ITT and AUC of mice fed a HFD at week 11, ***P<0.001. G, H) Values at the end of 100 d HFD: G) fasting blood glucose, *P<0.02; H) fasting serum insulin, *P<0.03. A two-tailed, unpaired t-test was used to determine statistical significance.

At the end of the LFD study, no significant difference in fasting blood glucose levels occurred between cGFJ group and control (Fig. 3A). In the fed state, cGFJ had no effect on serum insulin levels in mice fed a LFD (data not shown). cGFJ produced no significant differences in the GTT or ITT at either time (data not shown). Even without a high fat challenge, however, fasting serum insulin levels were 2-fold lower in the cGFJ *vs*. the control group fed a LFD (Fig. 3B).

At week 7 of the HFD, the GTT blood glucose area under the curve (AUC) was 6% lower (*P*<0.05) for the HFD-fed GFJ group compared to controls (data not shown). By week 11 this difference increased to 11% (Fig. 3C, D). In an initial ITT done at week 9, the blood glucose AUC was ∼17% lower (*P*<0.05) in the cGFJ group compared to controls (data not shown). This difference was maintained at week 13 (Fig. 3E, F). At the end of the HFD study, fasting blood glucose values were 13% lower in cGFJ mice compared to controls (Fig. 3G). Fasting serum insulin levels were 72% lower in the cGFJ group compared to controls (Fig. 3H). Fed insulin levels were not different between the two groups (data not shown).

**Figure 4:**
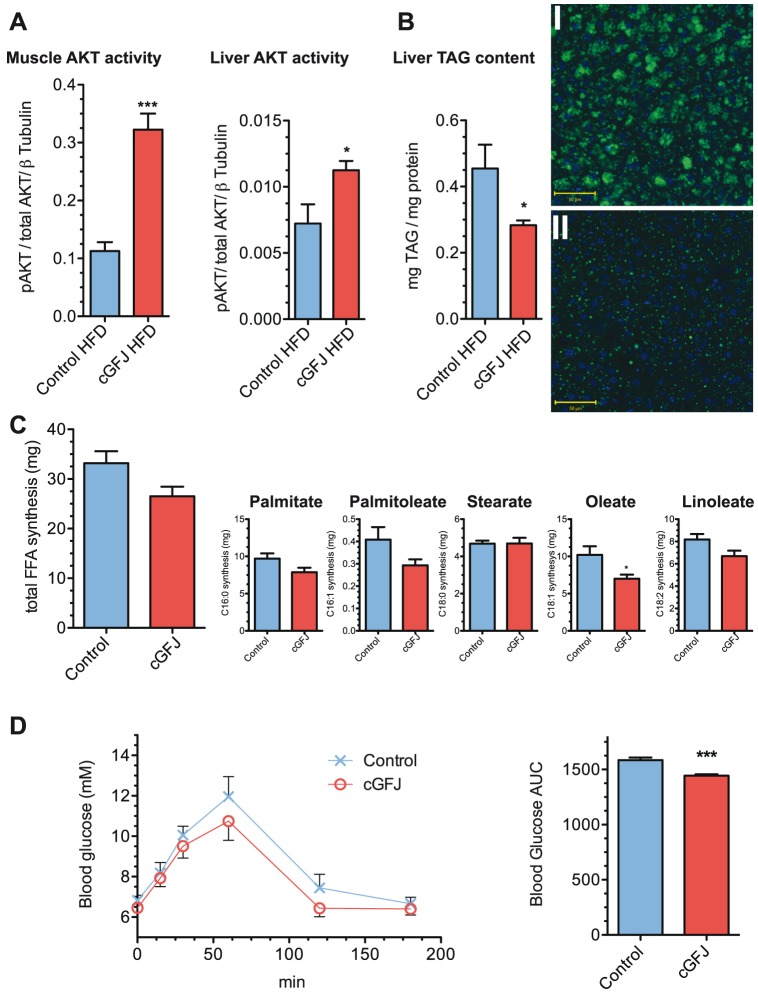
Impact of cGFJ on AKT activity in liver and skeletal muscle, TG content, and fatty acid synthesis. Mice were fed a HFD and 50% cGFJ for 100 d as described in the legend of Fig. 2. A) pAKT/total AKT ratios in muscle (***P = 0.0002) and liver (*P<0.05); B) liver TG, *P<0.05; I and II) Representative sections from control water and GFJ treated animals, respectively; C) total FFA synthesis and individual FFA; D) GTT and AUC at week 6, *P*<0.005. A two tailed, unpaired t-test was used to determine statistical significance.

Improved insulin sensitivity as a result of cGFJ supplementation was confirmed by evaluating activation of AKT, the insulin receptor downstream kinase, in fasted mice. GFJ produced a 3-fold and 1.4-fold increase, respectively in p-AKT/total AKT ratios in the quadricep muscle and liver, compared to controls (Fig. 4A).

Consumption of 50% cGFJ reduced the amount of TG in livers of mice fed a HFD by 38% compared to controls, and reduced the numbers and sizes of lipid droplets after ten days (Fig. 4B). Using an *in vivo* heavy water labeling approach [18], we determined that total fatty acid de novo synthesis in liver did not differ significantly between cGFJ and controls, and the synthesis rates of specific fatty acids were similar to control, except for oleate (Fig. 4C). In contrast to fatty acid synthesis, a PTT showed that the 10-day intervention in HFD-fed mice produced a 9% decrease in gluconeogenesis (Fig. 4D).

### GFJ improves metabolic variables after obesity onset

**Figure 5:**
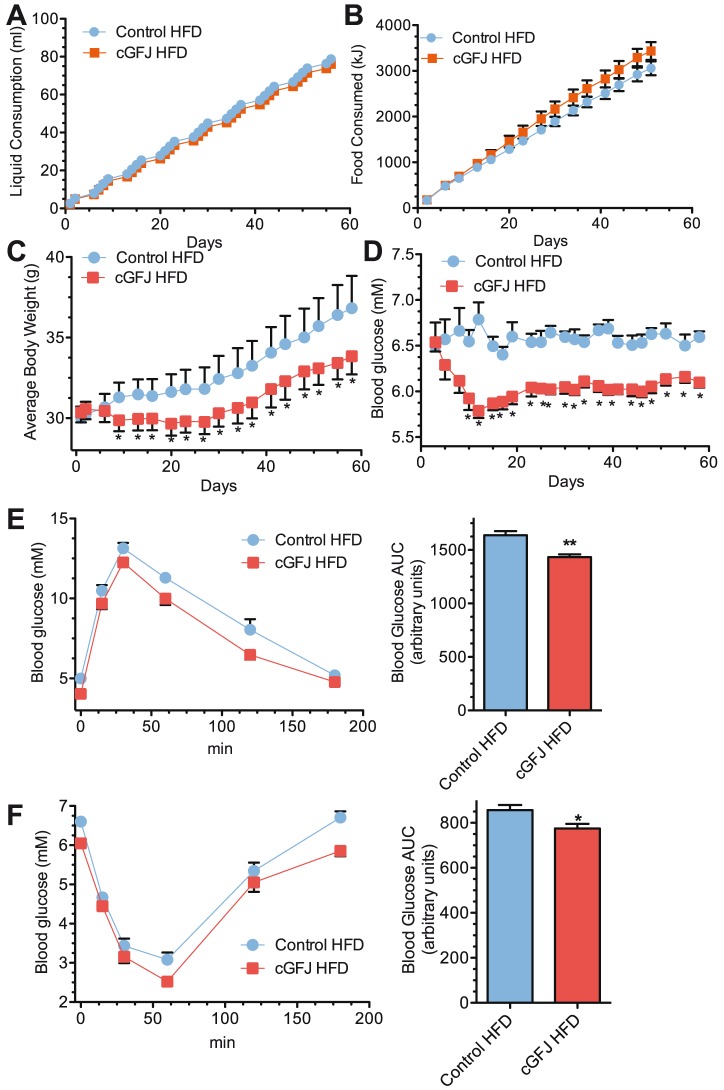
Impact of cGFJ on established diet-induced obesity. Mice were fed a HFD for 6 wk starting at 4 wk old. Animals were then divided randomly into control and GFJ groups (day 0) and HFD feeding was continued an additional 56 d: A) cumulative liquid consumption; B) cumulative food consumption; C) total body weights; D) blood glucose; E) GTT and AUC at week 6, *P*<0.002; F) ITT and AUC at week 7, *P*<0.03. A two tailed, unpaired t-test was used to determine statistical significance.

To determine the impact of cGFJ on mice with diet-induced obesity, animals were fed a HFD 10 wk and then allowed access to 50% GFJ, while continuing the HFD. Introduction of cGFJ did not change daily or cumulative liquid or calorie consumption (Fig. 5A, B). By the end of this experiment on day 55, the 50% cGFJ group weighed ∼8% less than controls (GFJ, 33.4±1 g vs. control, 36.4 g±1.9 g, *P*<0.05) (Fig. 5C). Body weights had become significantly different starting on day 9 (*P*<0.05). A 13% decrease in blood glucose occurred as early as day 10 post intervention (Fig. 5D). Final resting serum glucose levels in the cGFJ group were 110±1 mg/dL (96.1±1 mM) compared to the control value of 119±1 mg/dL (6.6±1 mM) (*P*<0.5). A GTT at week 6 post intervention revealed a 12.5% decrease (*P*<0.05) in the AUC, consistent with increased glucose tolerance (Fig. 5E). This observation was augmented by an ITT at week 7, which showed an AUC for the cGFJ group 9.5% lower than control (Fig. 5F).

### Unique metabolic effects of GFJ

**Figure 6:**
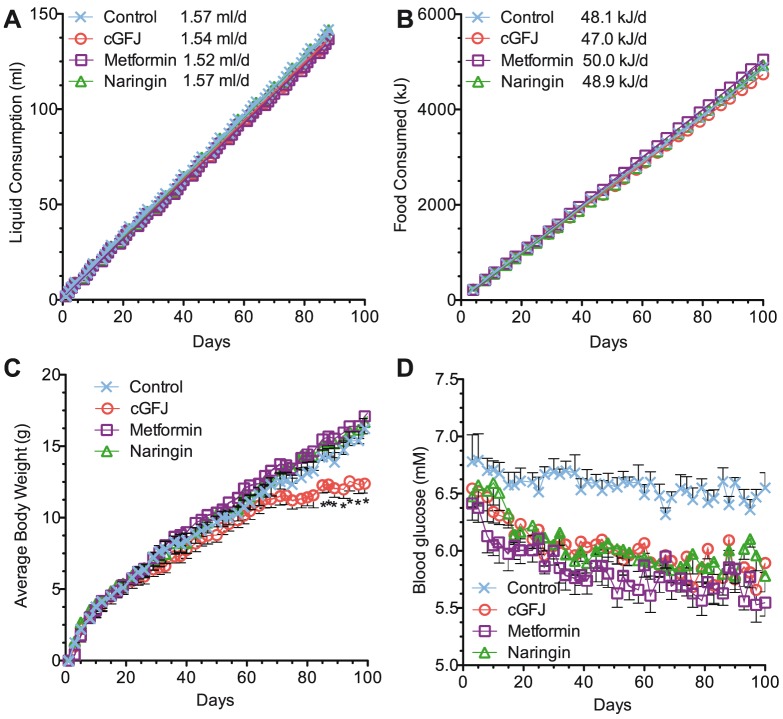
Comparison of metabolic effects of cGFJ, naringin, and metformin. Mice fed a HFD were given cGFJ, water containing naringin or metformin, or control water for 106 d: A) cumulative liquid consumption and rates based on linear regression. Slopes did not differ significantly; B) cumulative food consumption and rates based on linear regression. Slopes did not differ significantly; C) total body weights. 2way ANOVA with Bonferroni posttests showed that treatment and time had a significant effect (p<0.0001) on body weight. Bonferroni posttests only showed significant differences in body weight between the control and cGFJ group form 92 onward but not for any of the other groups; D) blood glucose. 2way ANOVA with Bonferroni posttests showed that treatment and time had a significant effect (p<0.0001) on blood glucose levels. Bonferroni posttests comparisons to the water control group showed significant differences for cGFJ and naringin starting day 27 and metformin starting day 8. Differences between cGFJ, naringin, and metformin were non-significant at all time points.

We compared the metabolic effects of GFJ with those of naringin, a bioactive compound in cGFJ, and metformin, a drug used widely to treat type 2 diabetes and nonalcoholic steatohepatosis during 106-day of feeding a HFD [19], [20]. Liquid and calorie consumption was comparable among all four groups (Fig. 6A, B). Body weights of the 50% cGFJ group, but not of the metformin- or naringin-supplemented groups, were significantly lower compared to controls at the end of the study (control, 32.9±0.5 vs. cGFJ, 28.2±g, *P*<0.05) (Fig. 6C). All three intervention groups had a statistically significant drop in blood glucose compared to controls by day 8 (113 to 110 mg/dL or 6.3 to 6.1 mM), which continued on days 10 (119 to 114 mg/dL or 6.6 to 6.3 mM) and 17 (117 to 111 mg/dL or 6.5 to 6.2 mM) (Fig. 6D). Blood glucose at the end of the study was ∼20% lower in the three treatment groups compared to the control.

The AUC of a GTT at week 7 was not significantly lower for the cGFJ, naringin, or metformin groups than the control group (data not shown). By week 13, however, the AUC values for the cGFJ, naringin, and metformin groups were 8, 7 and 12% lower than the control group, respectively (*P*<0.05 for all) (data not shown). An ITT at week 14 revealed AUC values 9, 8 and 15% lower for the GFJ, naringin, and metformin groups, respectively, relative to the control group (*P*<0.05 for all) (data not shown).

**Figure 7:**
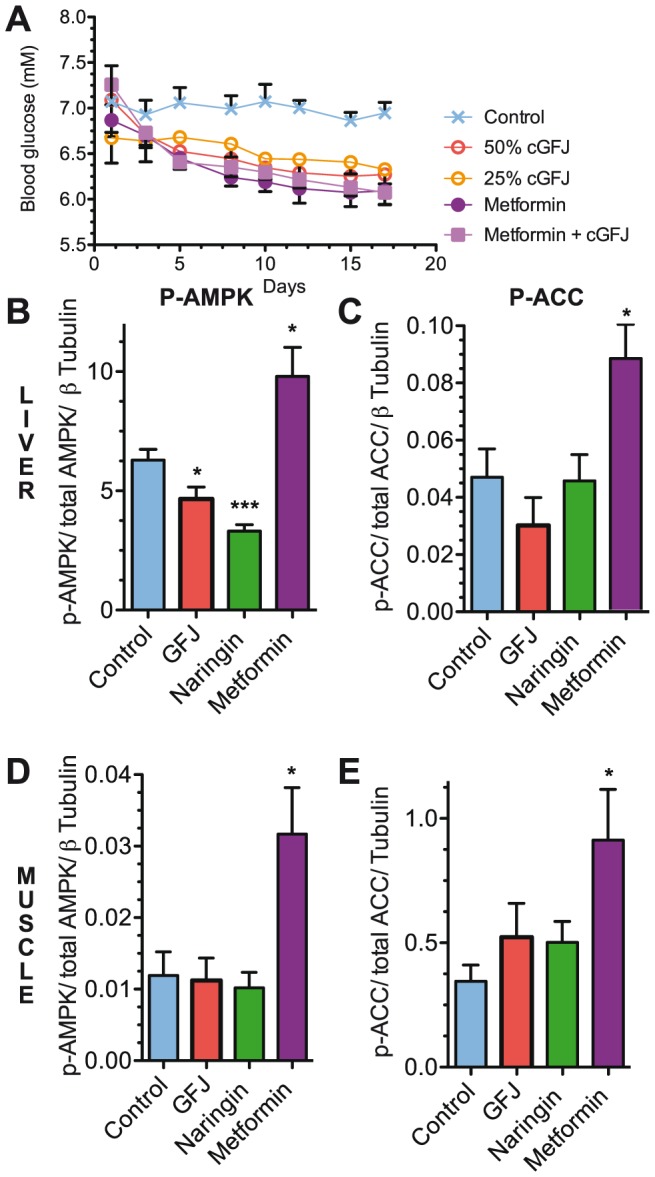
Effects of cGFJ on serum glucose levels and on AMPK and ACC phosphorylation in muscle and liver. Mice were fed a HFD. A) Blood glucose levels during 17 d access to 50% cGFJ, 25% cGFJ, metformin, metformin and 50% cGFJ, or water. B-E) Mice were allowed access to 50% GFJ, naringin or metformin in control water for 106 d starting at weaning: B) liver pAMPK/AMPK, *P<0.04, ***P = 0.0002; C) liver pACC/ACC, *P<0.03; D) muscle pAMPK/AMPK, *P<0.03; E) muscle pACC/ACC, *P*<0.03. Phospho- and total protein were determined by western blot and normalized to tubulin. A two-tailed, unpaired t-test was used to determine statistical significance.

To determine the possibility of synergistic or additive effects on blood glucose, mice fed a HFD were allowed access to metformin in a solution of 50% cGFJ for 17 d. In the same experiment a second group of mice was allowed access to 25% GFJ. The combination of metformin and 50% cGFJ produced no significantly different effect on blood glucose relative to either alone (Fig. 7A). Blood glucose in the 25% cGFJ group decreased comparably to the 50% cGFJ, metformin, and metformin plus 50% GFJ groups. The final blood glucose value of each treatment group was 11–14% lower than control (*P*<0.05).

### cGFJ acts through an AMPK-independent mechanism

We compared the effects of metformin, naringin and 50% cGFK on p-AMPK levels in liver and muscle of mice fed a HFD for 15 weeks. cGFJ decreased the ratio p-AMPK/AMPK in liver 25%, compared to a 52% decrease in response to naringin, and to a 1.6-fold increase in response to metformin (Fig. 7B). We assayed p-acetyl-CoA-carboxylase (ACC), because AMPK deactivates ACC via phosphorylation [21], [22]. p-ACC was unchanged in the GFJ and naringin groups, but metformin increased p-ACC in liver 2-fold (Fig. 7C). In muscle, only metformin increased p-AMPK (2.7-fold) (Fig. 7D) and p-ACC/AC (2.6-fold) (Fig 7E).

**Figure 8:**
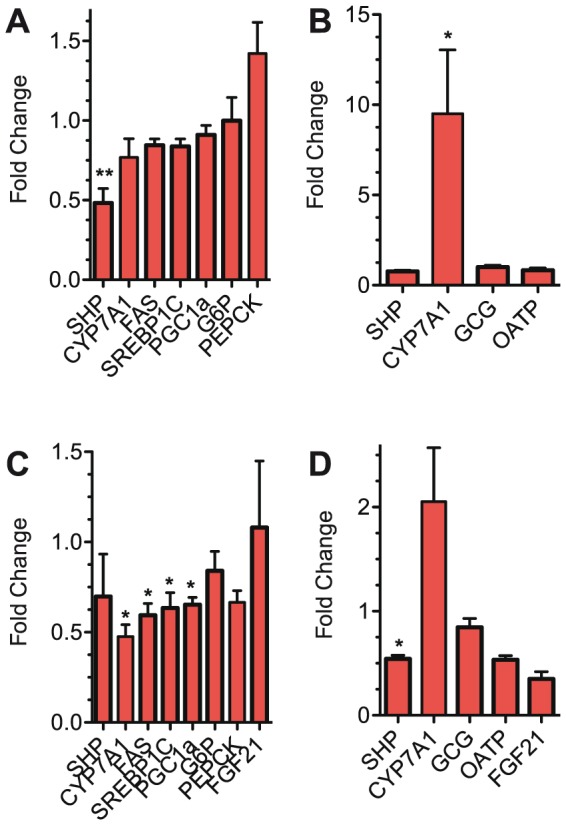
Gene expression changes caused by cGFJ. Mice were allowed to drink 50% cGFJ or control water for 17 or 100 d, as described in the legend of Fig. 2. A) liver, short term; B) small intestine, short term; C) liver, long term; D) small intestine, long term. Values were normalized to control values set as 1: *P<0.05.

In a further attempt to determine the mechanism(s) of cGFJ effects we assessed changes in expression of select metabolic genes in liver and small intestine of mice fed a HFD for 17 days (Fig. 8A, B) or after 100 d (Fig. 8C, D). In the short-term experiment, the only significant change in livers of mice treated with cGFJ was a 50% decrease in SHP, and the only significant change in intestine was a 9.5-fold increase in CYP7A1. In the long-term experiment, liver showed a 52% decrease in CYP7a1, a 41% decrease in FAS, a 37% decrease in SREBP1c, and a 35% decrease in PGC1α. In the small intestine, the only significant change was a 46% decrease in SHP.

## Discussion

We developed a well-controlled animal model, which showed that regardless of the amount of fat in the diet, consumption of cGFJ markedly lowered fasting serum insulin. In addition, consumption of 25% or 50% cGFJ reduced fasting glucose in mice fed a HFD, and 50% cGFJ reduced the rate of weight gain in mice fed a HFD. These outcomes did not depend on reduction of caloric uptake between cGFJ and control groups. The anti-glycemic effect of cGFJ occurred within five days, and was as pronounced as the effect of metformin, one of the most potent and widely-used anti-diabetic medications [23]. Although synergistic effects were not observed between GFJ and metformin, the two appear to act through different mechanisms, because metformin activated AMPK and canonical downstream signaling pathways in liver and muscle (p-ACC), whereas cGFJ decreased AMPK phosphorylation (liver) or had no significant effect (muscle)—a result reflected in unchanged p-ACC levels. Regardless, drinking centrifuged (pulp-free) GFJ corresponding to ∼3.5–4 cups (830–950 ml) per day for an average 70 kg human adult, had robust hypoglycemic effects in mice fed a HFD, warranting further study of its health-promoting effects, identification of bioactive components, and mechanisms of action.

cGFJ behaved similarly, but not identically, to one of its bioactive compounds, naringin, which lowered blood glucose levels of HFD-fed animals without altering the activity of AMPK or ACC. This latter finding differs from results of Pu et al. [8], who reported robust activation of AMPK and inactivation of ACC in livers of HFD-fed C57Bl/6J mice in response to naringin, with comparable naringin doses, albeit presented in the diet, instead of in the drinking medium. We did not find that naringin caused weight loss or suppressed expression of the hepatic gluconeogenic enzymes PEPCK and G6Pase, as reported by Pu. The composition of the HFD used here differed from that of Pu (% J from fat/carbohydrate/protein, 60/20/20 vs. 37/43/20, respectively). Nevertheless, our results are consistent with reports that naringin has hypoglycemic, but not weight lowering effects [6], [24]. It should be noted for practical reasons we used cGFJ throughout the study to avoid clogging liquid intake monitors. Whether pulp-containing GFJ would have enhanced or reduced metabolic effects remains to be determined, but the fact remains that GFJ contains a compound or compounds other than naringin with health-promoting properties.

We were unable to identify the proximate mechanism(s) of cGFJ effects. Possibly, subtle but cumulative differences in caloric absorption, respiration rates, or anti-inflammatory properties contribute to the phenotype. This possibility is supported by a need for 78 days of a HFD before weight differences induced by cGFJ became statistically significant [24]. Both the sweetened water control and cGFJ were acidic, but cGFJ had a lower pH at 3.5. All ingested liquids had to pass through a range of robust intraluminal pH gradients from the stomach (pH 1–3) to the small intestine (pH 6–7.4) [25], and it is unlikely that cGFJ consumption would alter duodenal pH to a degree that would impact pancreatic enzyme function or nutrient absorption. This conclusion is supported by the similarity in caloric value of feces collected over the final 24 hr of the 100-day-study from the cGFJ and control groups, and the lack of differences in absorption of glucose, oleic acid or taurocholic acid between cGFJ and the control mice.

Interestingly, cGFJ decreased expression of the small heterodimer partner (SHP), which antagonizes function of multiple nuclear hormone receptors that regulate intermediary metabolism, such as LXRα, RARα, and PPARγ [26] [27]. Down regulation of Cyp7a1, FAS, SREBP1c, and PGC1α also are consistent with multiple alterations in lipid homeostasis [28], [29]. These data imply that cGFJ alters regulation of fat synthesis and storage.

Potential benefits should be evaluated in context of reports that GF and GFJ components interact with several proteins that catalyze drug metabolism and absorption, and may cause health issues by modifying drug potency [30]. Many studies have shown that GF or GFJ, or their components alter drug pharmacokinetics, but altered pharmacokinetics doesn't necessarily alter pharmacodynamics [31]. In the ∼24 years since the potential for GFJ/GF consumption to alter drug potency was proposed, less than a dozen case reports have correlated GF or GFJ consumption with adverse clinical outcomes [10]. In most, if not all, the amount of GFJ consumed did not reflect normal consumption [32], [33], associations between GFJ and clinical manifestations were correlative [34], and patients had either severe pre-existing illnesses and/or confounding factors [35]. The possibility that excessive GFJ consumption could cause health issues in a select population taking specific drugs should not be dismissed, but nor is it appropriate to extrapolate these limited observations to the general population. A critical and evidence-based assessment of the potential beneficial vs. harmful effects of GF and GFJ consumption seems prudent.

We have provided new evidence for potential health promoting properties of GFJ in murine HFD-driven obesity and non-obesity models. These results justify additional studies in animal models and humans to assess the mechanisms and scope of GFJ action.
